# Emerging Roles of SIRT3 in Cardiac Metabolism

**DOI:** 10.3389/fcvm.2022.850340

**Published:** 2022-03-18

**Authors:** Krishnega Murugasamy, Aastha Munjal, Nagalingam Ravi Sundaresan

**Affiliations:** Cardiovascular and Muscle Research Laboratory, Department of Microbiology and Cell Biology, Indian Institute of Science, Bengaluru, India

**Keywords:** SIRT3, metabolism, glycolysis, mitochondrial oxidation, heart failure

## Abstract

The heart is a highly metabolically active organ that predominantly utilizes fatty acids as an energy substrate. The heart also derives some part of its energy by oxidation of other substrates, including glucose, lactose, amino acids and ketones. The critical feature of cardiac pathology is metabolic remodeling and loss of metabolic flexibility. Sirtuin 3 (SIRT3) is one of the seven mammalian sirtuins (SIRT1 to SIRT7), with NAD^+^ dependent deacetylase activity. SIRT3 is expressed in high levels in healthy hearts but downregulated in the aged or diseased hearts. Experimental evidence shows that increasing SIRT3 levels or activity can ameliorate several cardiac pathologies. The primary deacetylation targets of SIRT3 are mitochondrial proteins, most of which are involved in energy metabolism. Thus, SIRT3 improves cardiac health by modulating cardiac energetics. In this review, we discuss the essential role of SIRT3 in regulating cardiac metabolism in the context of physiology and pathology. Specifically, we summarize the recent advancements that emphasize the critical role of SIRT3 as a master regulator of cardiac metabolism. We also present a comprehensive view of all known activators of SIRT3, and elaborate on their therapeutic potential to ameliorate energetic abnormalities in various cardiac pathologies.

## Introduction

The heart has one of the highest metabolic rates of any organ (1). In the physiological state, an adult heart exhibits metabolic flexibility and derives large proportion of its energy from fatty acids (70%), glucose and lactose (20–30%) (2, 3). Amino acid and ketone metabolism also contribute to the ATP pools in the heart (4). Heart failure is a pathological condition marked by reduced cardiac output and impaired cardiac energetics (5). Various cardiac pathologies serve as the underlying cause of heart failure, including hypertension, diabetic cardiomyopathy, and ischemic heart disease. These conditions are characterized with changes in cardiac metabolism that contribute to the development of an energy deficit and culminate in heart failure (6). In conditions of idiopathic dilated cardiomyopathy and pressure overload-induced heart failure, fatty acid metabolism is reduced due to downregulation of enzymes involved in fatty acid metabolism and imbalance in intracellular triglyceride turn over. Under such conditions the heart exhibits enhanced glycolysis as a compensatory mechanism (7–9). Conversely, in diabetic cardiomyopathy, the heart relies extensively on fatty acids, due to increased plasma fatty acid levels and develops insulin resistance which renders the heart more susceptible to ischemia (10). Studies also suggest that the post translational modification of proteins involved in cellular metabolism is emerging as a key regulatory step in modulating cardiac physiology and pathology (11).

Sirtuins are Class III histone deacetylases (HDACs) characterized by NAD^+^ dependent enzymatic activity. The first sirtuin, silent mating type information regulator (Sir2), was identified in yeast and has since been shown to mediate longevity benefits of calorie restriction in several organisms, including *C. elegans* and *D. melanogaster* (12–14). In mammals, seven orthologs of Sir2 (SIRT 1-7) are identified (15). Each mammalian isoform of sirtuins is known to differ in its subcellular localization, target and activity. While SIRT1, SIRT6 and SIRT7 are predominantly nuclear among the seven sirtuins, SIRT2 is cytosolic. In mitochondria, SIRT3, SIRT4, and SIRT5 are predominant sirtuin isoforms. However, SIRT1, SIRT2, and SIRT7 are known to shuttle between cytoplasm and nucleus based on their activation status and regulate various histone and non-histone proteins (16–23). Similarly, SIRT3 and SIRT5 are also found in the cytoplasm and nucleus (24–27). SIRT6, although predominantly nuclear, has been observed to localize to cytoplasmic stress granules in response to stress (21). Besides well-characterized, classical deacetylase activity, some sirtuins exhibit deacylase (SIRT1, SIRT2, SIRT4, SIRT6) (28), lipoamidase (SIRT4), ADP-ribosyltransferase (SIRT4, SIRT6), depalmitoylase (SIRT6), desuccinylase (SIRT5), demalonylase (SIRT5) and deglutarylase (SIRT5) activity (29–35).

SIRT3 is a mitochondrial deacetylase expressed at high levels in metabolically active organs such as the brain, kidney, liver, heart and brown adipose tissue (36). SIRT3 regulates several cellular processes, including mitochondrial DNA damage repair, gene expression, bioenergetics, redox balance, autophagy and apoptosis (26, 37–42) (Table 1). SIRT3 regulates mtDNA repair by interacting with the DNA repair enzyme 8-oxoguanine-DNA glycosylase 1 (OGG1) and positively regulating its incision activity and turnover. This has been proposed to blunt genotoxicity-induced apoptosis in γ-irradiated cells (40). In addition to mtDNA repair, SIRT3 also regulates mitochondrial dynamics, a mitochondrial process that is key to overall mitochondrial function, including mitochondrial metabolism. During pathological cardiac stress, the inner mitochondrial membrane fusion protein OPA1 is hyperacetylated. In this state, it is characterized with reduced GTPase activity. SIRT3-mediated deacetylation of OPA1 has been shown to promote its GTPase activity and augment mitochondrial fusion (44). Furthermore, under oxidative stress, SIRT3 modulates mitochondrial mass by upregulating mitochondrial fission proteins dynamin-related protein 1 (DRP1) and fission protein 1 (Fis1) *via* FOXO3 deacetylation (43). In addition to this, SIRT3 mediates the longevity benefits of caloric restriction by deacetylating molecular targets involved in mitochondrial maintenance and metabolism (54, 55). Furthermore, clinical studies show that exercise-mediated rescue of metabolic disorder is associated with SIRT3 upregulation (56). In mice, swimming exercise results in increased levels of SIRT3 short form and physiological cardiac hypertrophy, characterized by increase in cardiomyocyte size without fibrosis or pathological remodeling (38). However, mice subjected to chronic infusion of isoproterenol or 6 weeks of aortic banding show marked reduction in SIRT3 short isoform and develop severe pathological hypertrophy with increased fibrosis (38). While SIRT3 knock-out (KO) mice show severe adverse remodeling, SIRT3 overexpressing mice show protection against adverse remodeling induced by hypertrophic agonists (26).

**Table 1:** SIRT3 targets and their biological functions.

**Molecular targets**	**Biological function**	**References**
FOXO3a, OPA1	Mitochondrial dynamics	(43, 44)
OGG1	Mitochondrial DNA repair	(40)
MnSOD	ROS homeostasis	(45)
Ku70	Cell Survival	(26)
CypD	Mitochondrial structure and function	(46)
p53	Glucose metabolism	(47)
PDC	Glucose metabolism	(48)
TCA enzymes (citrate synthase, aconitase, isocitrate dehydrogenase, succinate dehydrogenase, malate dehydrogenase)	Glucose metabolism	(39, 49–52)
ETC enzymes (succinate dehydrogenase, NDUFA9 subunit of complex I)	ATP production	(37)
HMG Co-A synthase	Ketogenesis	(53)
Glutamate dehydrogenase	Amino acid metabolism	(36)

Whole-body SIRT3-KO mice show signs of cardiac hypertrophy and interstitial fibrosis by 8 weeks and exhibit a 19% reduction in lifespan (38, 57). Although these mice appear normal under physiological conditions, they are predisposed to multiple pathologies upon aging or under stress conditions—indicating that SIRT3 may be involved in preserving cardiac function by restoring cardiac energetics and conserving metabolic flexibility of the heart. Upon aging, whole body SIRT3-KO mice show adverse cardiac remodeling, with subtle abnormalities in the liver, kidney and brain (57). While tissue-specific SIRT3 ablation in liver, muscle or brown adipose tissue does not recapitulate the germline SIRT3-KO phenotype (58, 59), the hearts of cardiac-specific SIRT3-KO mice resemble aged hearts (41). Typical characteristics of an aging heart include cardiac hypertrophy, insulin resistance, myocyte loss and cardiac fibrosis. SIRT3 has been shown to regulate each of these processes (Table 2). The hearts of whole body SIRT3-KO mice exhibit cardiac hypertrophy, interstitial fibrosis, contractile dysfunction and inflammation (42). In response to haemodynamic stress such as pressure overload, the hearts of SIRT3-KO mice display adverse cardiac remodeling and enhanced cardiomyocyte apoptosis (26, 38, 41, 68). Further, post myocardial infarction, these mice suffer from impaired angiogenesis (69–71). Under high fat diet or diabetic conditions, SIRT3-KO mice show increased susceptibility to insulin resistance (67, 72). At the cellular level, these mice are characterized with redox imbalance, impaired metabolism, mitochondrial dysfunction and defective autophagy, recapitulating key features of an aged heart (41). At the molecular level, SIRT3-KO mice are characterized by hyperacetylation of mitochondrial proteins (36, 73). Most of these proteins are involved in energy metabolism. These mice also show more than 50% reduction in ATP pools (37, 68). It is a general understanding that SIRT3's deacetylase activity plays a major protective role against heart failure by regulating cardiac ATP levels (74). However, a recent study employing a carnitine acetyltransferase/Sirt3 double knock-out model argues that hyperacetylation of the mitochondrial proteome alone does not culminate in heart failure (75). This is an unexpected finding that may indicate the presence of other compensatory mechanisms for regulation of metabolic flexibility in the heart.

**Table 2:** SIRT3 targets in aging-associated cardiac pathophysiology.

**Aging-associated phenotype**	**Model system**	**SIRT3 Molecular target**	**Downstream effect**	**References**
Hypertrophy	Whole body SIRT3-KO mice	FOXO3a	Catalase activity (↑) MnSOD activity (↑)	(38)
	Pressure overload by abdominal aortic banding in Wild type mice	LKB1	mtUPR response (↑); Fatty acid metabolism (↑); Ketone metabolism (↑)	(60)
Cardiac fibrosis	Rat neonatal cardiomyocytes; Whole body SIRT3-KO mice	H3K27	FOS expression (↓); Inflammatory and fibrotic response (↓)	(42)
	HFD-fed whole body SIRT3-KO mice	Not known	ROS levels (↓), NFKb-MCP-1 activity (↓), Macrophage infiltration (↓), Cardiac fibrosis (↓)	(61)
	AngII-treated whole body SIRT3-KO mice; SIRT3 transgenic mice	GSK3β	Phosphorylation and degradation of Smad 3 and β-catenin (↑), Cardiac fibrosis (↓)	(62)
	Neonatal rat fibroblasts	Not known	PPARγ expression and activity (↑), β-catenin degradation (↑), Cardiac fibrosis (↓)	(63)
	Fibroblasts from whole body SIRT3-KO mice	STAT3	NFATc2 expression (↓), Cardiac fibrosis (↓)	(64)
Cardiomyocyte loss	SIRT3 overexpressing cardiomyocytes	OPA1	L-OPA to S-OPA form conversion (↓), Apoptosis (↓)	(44)
	H9C2 cardiomyocyte overexpressing SIRT3	Not known	H_2_O_2_ levels (↓), NFkB activity (↑), Bcl2/Bax ratio (↑), Apoptosis (↓)	(65)
	SIRT3 overexpressing rat neonatal cardiomyocytes	Ku70	Bax sequestration (↑), Apoptosis (↓)	(26)
	Cardiac-specific SIRT3-KO mice	p53	Parkin activity (↑), mitophagy (↑)	(41)
	Neonatal mice cardiomyocytes	Not known	AMPK activity (↑), Mitochondrial biogenesis (↑)	(66)
Insulin resistance	HFD-fed whole-body SIRT3-KO mice; Human umbilical vein endothelial cells	Not known	mtROS production (↓), eNOS activity (↑), NO production (↑), Insulin sensitivity (↑)	(67)

SIRT3 overexpression has been noted to rescue a range of cardiopathology phenotypes by improving the metabolic flexibility of cardiomyocytes (47, 76). In this review, we summarize the molecular targets of SIRT3 involved in energy metabolism and elaborate on the underlying regulatory mechanisms observed in physiological and pathological cardiac metabolism. We also present a comprehensive view of all known modulators of SIRT3 activity and elaborate on their therapeutic potential to ameliorate energetic abnormalities in various cardiac pathologies.

## Regulation of SIRT3 Expression in the Heart

The human *SIRT3* gene is located in chromosome region 11p15.5. It shares a bidirectional promoter with 26S proteasome non-ATPase subunit 13 (*PSMD13*) (77, 78). Haplotype studies have revealed that both the genes are located in the chromosomal region that is associated with the longevity. Furthermore, the common promoter region contains Sp1 sites for transcriptional regulation of the two genes. Together these results indicate that *SIRT3* and *PSMD13* may be functionally linked and co-regulated (78). The shared promoter region also contains binding sites for GATAs, NF-kB, ZF5, Activator protein (AP-1), and specificity protein-1 (Sp-1) (78). However, their role in the transcriptional regulation of SIRT3 expression remains to be established conclusively. On the other hand, coactivator peroxisome proliferator-activated receptor γ coactivator 1-α (PGC-1α) has been well explored as a transcription regulator of SIRT3 (79). During energetic stress, PGC-1α co-localizes with estrogen-related receptor-α (ERRα) on the ERR binding element (ERRE) in the mSirt3 promoter (79). PGC-1α also binds with and co-activates the transcription factor, nuclear respiratory factor 2 (NRF2). An *in vitro* study reported NRF2 as a positive regulator of SIRT3. Since the PGC-1α/NRF2 axis is activated upon dietary restriction, NRF2-mediated upregulation of SIRT3 is expected to occur under nutrient stress—where it may be required to enhance ATP production (80). A post-transcriptional regulator of SIRT3, microRNA-195 binds to 3′ untranslated region of mRNA and downregulates SIRT3 expression (81, 82). At the post-translational level, cAMP directly binds to and stabilizes SIRT3. Since cAMP levels are upregulated during starvation, it may be expected to activate SIRT3 during nutrient stress (83).

Cardiac SIRT3 expression fluctuates with various physiological factors, including age, diet, and exercise. Notably, exercise positively modulates SIRT3 expression in the heart (84, 85). Aging, on the other hand, is reported to have gender-specific effects on cardiac SIRT3 expression. SIRT3 levels don't vary significantly between young and older males. However, there is a pronounced reduction in SIRT3 expression in female hearts with age (86). Caloric restriction has been reported to ameliorate aging dependent decrease in SIRT3 levels (87). High-fat diet reduces cardiac SIRT3 levels (88). Although the mechanism is unclear, *in vitro* studies have demonstrated that ROS overload is linked with decreased SIRT3 mRNA and protein expression (65).

The levels of SIRT3 is also decreased in pathological conditions. For instance, in ischemia or hypoxia, HIF-1α upregulates the expression of Wnt3a, which in turn negatively regulates SIRT3 expression (89). In contrast, during diabetes, the initial stages of cardiac hypertrophy are marked with elevated SIRT3 expression. However, the levels eventually decline when the pathological state progresses to diabetic cardiomyopathy or heart failure (88, 90–92). In addition to these factors, metabolites have also been shown to function as regulators of SIRT3 expression. For instance, ketone body induced upregulation of SIRT3 has been observed in cultured human fibroblasts under oxidative stress (93). Interestingly, SIRT3 is also regulated by other sirtuins. Studies in several cell lines, including HeLa, HEK, C2C12, show that SIRT1 negatively regulates the transcription of SIRT3 by deacetylating ZF5, a transcriptional repressor that sequesters the SIRT3 transcription factor, SP1 (94). In contrast, other studies report SIRT1 as a positive regulator of SIRT3 in rat hearts, where it deacetylates and activates PGC-1α (95). Most recently, the SIRT1-PGC-1α-NRF1-SIRT3 signaling axis has been implicated in amelioration of mitochondrial dysfunction, and insulin-resistance in high fructose diet-fed rats (95). Additionally, SIRT3 is also a direct deacetylation target of SIRT1. In aged and obese mice, SIRT1 expression is reduced, and SIRT3 is hyperacetylated. Acetylation of SIRT3 at K57 culminates in loss of its deacetylation activity, and triggers proteasomal degradation. The authors propose that SIRT1-mediated deacetylation of SIRT3 can restore its activity and rescue metabolic dysfunction in livers of obese mice (96). SIRT6 is another sirtuin known to regulate SIRT3. It enhances SIRT3 expression by downregulating the expression of kelch-like-ECH-associated protein 1 (Keap1), a protein that binds to and sequesters the SIRT3 transcription factor, Nrf2. Furthermore, SIRT6 also binds to and stabilizes Nrf2—ultimately upregulating the expression of SIRT3 in the heart (97).

## Subcellular Localization of SIRT3

The localization of SIRT3 is heavily debated. The human SIRT3 (hSIRT3) exists as two isoforms. The longer isoform is a 44kDa long, full-length protein localized in the cytoplasm and nucleus (98). It carries an N-terminal mitochondrial localization sequence (MLS) which is cleaved by the matrix processing peptidase (MPP) in the mitochondria (77). Proteolytic processing of the long isoform yields a shorter, 28 kDa long isoform of hSIRT3 that acts as a functionally active mitochondrial deacetylase (24, 77).

Similarly, different isoforms for murine SIRT3 (mSIRT3) are also known. A study in 3T3 fibroblasts showed that alternative splicing of the murine *SIRT3* gene results in three protein variants, M1, M2, and M3 (99). The variants M1 and M2 are tagged with an MLS. M1 and M2, when in the cytoplasm and nucleus, are full-length long isoforms. However, upon translocation into mitochondria, they are truncated by proteolytic cleavage of the MLS. Unlike M1 and M2, the M3 splice variant is originally shorter and does not undergo proteolytic processing. Further, it lacks an MLS but carries an internal mitochondrial targeting sequence (MTS) and nuclear localizing signal (NLS) (99, 100). Reports involving mSIRT3 overexpression indicate that M3 is localized exclusively in the cytoplasm and nucleus of 3T3 fibroblasts. In contrast, more recent studies in these cells reflect that although it is localized predominantly in the cytoplasm and nucleus, it also partially localizes in mitochondria (101).

In murine hearts, endogenous SIRT3 has been reported in nuclear, cytoplasmic and mitochondrial fractions. Early reports from adult mouse hearts indicated that the long isoform (44 kDa) of SIRT3 is localized in the nucleus, cytosol and mitochondria. Meanwhile, the short isoform (28 kDa) was observed exclusively in mitochondria (26). Interestingly, in another study investigating isoform localization in the cardiomyocyte cell line H9C2, both isoforms were shown to localize in the mitochondria and the nucleus (102). Further, in these cells, the long isoform is more abundant in mitochondria when compared with the short isoform. In the same study, subcellular localization of the two isoforms was also evaluated upon SIRT3 overexpression in HEK293 cells. The long isoform was observed to localize majorly in the mitochondria, and at higher concentrations, in the cytoplasm. On the other hand, the short isoform localized exclusively in the cytoplasm. Together, these studies indicate that the subcellular localization of the isoforms may be highly cell-type specific (102).

## SIRT3 in Fatty Acid Metabolism

In the physiological state, nearly 70–80% of the cardiac energy demand is met by fatty acid metabolism (2). The adult heart relies primarily on fatty acid oxidation for sustained generation of ATP (2). Circulating free fatty acids are transported into the myocardium, in part, by passive diffusion across the plasma membrane. In addition to this, fatty acid uptake is regulated by fatty acid transporter protein (FATP), and fatty acid translocase CD36 expressed on endothelial cells and cardiomyocytes (103). Since the heart has a limited reservoir of triacylglycerol, continuous uptake of fatty acids ensures fuel availability for β-oxidation based energy generation.

The hypertrophic heart is characterized with diminished fatty acid metabolism (104). Activation of SIRT3 in hypertrophic heart has been shown to alleviate cardiac fibrosis, and ameliorate hypertrophy *via* SIRT3-mediated deacetylation and activation of LKB1. This in turn activates anti-hypertrophic LKB1-AMPK signaling in this hearts (105). Independently, activated AMPK has been shown to enhance expression of fatty acid transporters, CD36 and CPT1B in hypertrophic hearts (60, 105). Furthermore, activated AMPK is also known to enhance β-oxidation by downregulating the expression of malonyl-CoA, a negative regulator of fatty acid catabolism. AMPK achieves this by phosphorylating two key enzymes involved in the malonyl-CoA synthesis, viz. acetyl-CoA carboxylase (ACC) and malonyl-CoA decarboxylase (MCD) (106). Thus, the net result of SIRT3/AMPK activation is enhanced β oxidation. Since impaired energetics contribute to the development of cardiac hypertrophy, activating SIRT3-AMPK signaling may, at least in part, ameliorate myocardial metabolic dysfunction in hypertrophic hearts. On the other hand, the effect of AMPK activation in ischemic hearts is heavily debated. While few studies suggest that AMPK activation in ischemia is beneficial due to increased ATP production during oxygen insufficiency; others point that activated AMPK-mediated enhancement of β-oxidation triggers a decrease in glucose oxidation through the Randle cycle—resulting in uncoupling of glycolysis and glucose oxidation and ultimately stumping cardiac efficiency during reperfusion (107, 108). It is important to note that, currently, the effect of SIRT3-mediated AMPK activation on fatty acid metabolism in ischemic heart disease remains poorly understood.

One of the earliest known targets of SIRT3 is long-chain Acyl-CoA dehydrogenase (LCAD), the enzyme that catalyses the first step of the β-oxidation. SIRT3-KO mice possess high endogenous levels of acetylated LCAD and reduced fatty acid oxidation in several tissues, including the heart (96, 109). Furthermore, increased LCAD acetylation and reduced SIRT3 expression is also evident in rat models of heart failure (49). Moreover, in models of SIRT3 overexpression, SIRT3-mediated deacetylation of LCAD has been shown to increase fatty acid metabolism (110). Interestingly, under conditions conditions of high fat diet (HFD), a positive correlation has been reported between acetylation levels of LCAD and fatty acid oxidation. Notably, SIRT3-KO mice display increased acetylation and activity of LCAD under HFD (111). Furthermore, HFD increases the levels of GCN5L1, a mitochondrial acetyl transferase that increases acetylation and activation of mitochondrial enzymes (112). A recent report shows that under HFD conditions, cardiomyocyte specific GCN5L1-KO mice have reduced LCAD acetylation and activity in the heart, with no significant difference in SIRT3 levels between wild type and GCN5L1-KO mice. Similarly, other studies have also shown that the maturation of heart after birth to fatty acid metabolism is dependent on GCN5L1 levels and acetylation of LCAD, independent of SIRT3 (113, 114). Though SIRT3 and GCN5L1 have opposing effects on the acetylation status of LCAD, SIRT3 and GCN5L1 has been shown independently to have a positive effect on LCAD activity in heart. It is possible that acetylation status of specific residues rather than the protein itself influence its enzyme activity. While studies have characterized LCAD Lys-318 and Lys-322 as target residues for SIRT3 deacetylation (115), the LCAD residue targets for GCN5L1 remain to be characterized. Interestingly, increase in GCN5L1 expression and acetylation levels are linked with negative regulation of fatty acid oxidation in the liver (116). This difference between hepatic and cardiac metabolic regulation may arise due to tissue-specific fate of fatty acid oxidation. While fatty oxidation in the liver provides acetyl-CoA substrates for ketogenesis, it is utilized for oxidative phosphorylation in the heart. Inhibiting fatty acid enzymes by acetylation in the heart is expected to result in negative feedback loop disrupting the cardiac energy metabolism and function (116).

From studies in liver, SIRT3 is also known to directly deacetylate and regulate the mitochondrial trifunctional protein (TFP). The TFP is anchored to the inner mitochondrial membrane and comprises of 3 enzymes that catalyse the next three steps of β-oxidation. These include 2-enoyl coenzyme A hydratase (ECH), long-chain 3-hydroxy acyl-coenzyme A dehydrogenase (HAD) and long-chain 3-ketoacyl-CoA thiolase (KT). SIRT3 overexpression has been reported to rescue the TFA^+/−^ phenotype in hepatocytes (117). Although post-translational modification of TFA has not been studied extensively in the heart, there exists a strong positive correlation between SIRT3 levels, the deacetylation status of β-oxidation enzymes and fatty acid oxidation in cardiomyocytes and other tissues (109, 117).

**Figure 1:**
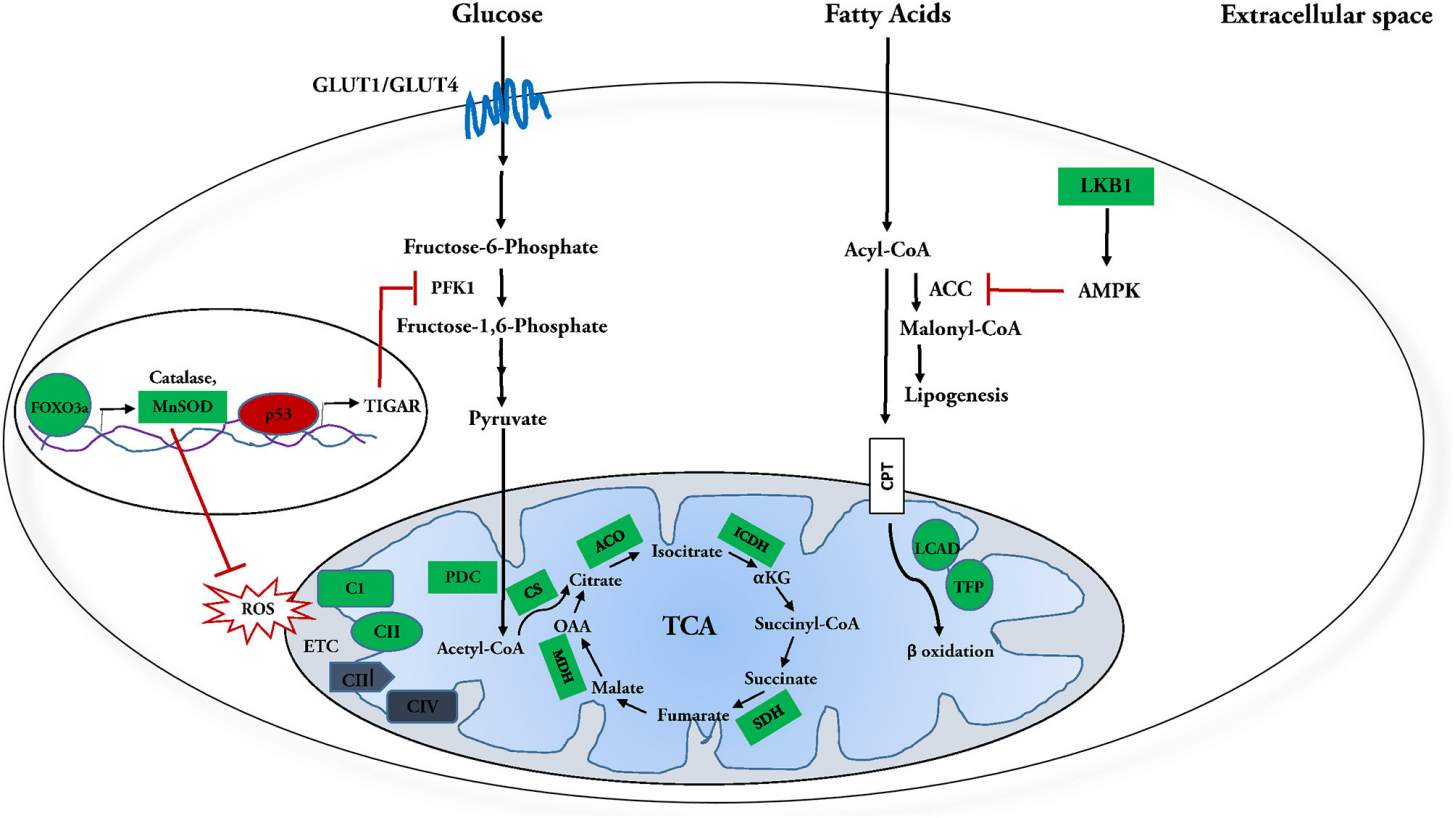
Molecular targets of SIRT3 in glucose and fatty acid metabolism. ***SIRT3 and*** ***fatty acid metabolism:*** SIRT3 deacetylates and activates enzymes involved in fatty acid oxidation, including long chain aycl-CoA dehydrogenase (LCAD) and trifunctional mitochondrial protein (TFP). Meanwhile it inhibits fatty acid synthesis by deacetylating and activating its inhibitor, LKB1, which in turn activates AMPK. AMPK further phosphorylates and inhibits Acetyl-CoA carboxylase (ACC) and Malonyl-CoA decarboxylase (MCD), reducing synthesis of malonyl-CoA, a negative regulator of fatty acid oxidation. In this manner, SIRT3 regulation culminates in enhanced fatty acid catabolism. ***SIRT3 and glucose metabolism:*** SIRT3 attenuates-activation of FOXO3a, which in turn transcriptionally upregulates ROS detoxification enzymes manganese dependent super oxide dismutase (MnSOD) and catalase. SIRT3 also directly interacts with and activates MnSOD. Attenuation of ROS inhibits HIF-1α from upregulating glycolytic genes during normoxia. SIRT3 enhances phosphofructokinase 1 (PFK 1) activity and subsequently upregulates glycolysis by increasing PFKFB3 activity via deacetylation-inactivation of p53 and subsequent suppression of its downstream target TIGAR. SIRT3 further increases glucose oxidation by activating pyruvate dehydrogenase complex (PDC) and targeting the enzymes involved in the tricarboxylic acid (TCA) cycle. SIRT3 mediated deacetylation of complex I subunit, NDUFA9 and succinate dehydrogenase (SDH) is necessary for efficient oxidative phosphorylation. In this manner, SIRT3 regulation culminates in enhanced utilization of glucose. Positively regulated SIRT3 molecular targets are indicated in green; negatively regulated targets are indicated in red.

SIRT3 has also been shown to improve fatty acid metabolism in calf hepatocytes treated with non-esterified fatty acids (NEFA). Overexpression of SIRT3 causes transcriptional downregulation of fatty acid synthesis enzymes acetyl-CoA carboxylase (ACC) and fatty acid synthase (FAS) in these cells. This is accompanied by the upregulation of enzymes involved in fatty acid oxidation, including CPT enzymes CPT1A, CPT2, and acetyl CoA oxidase (118). Similarly, the hearts of SIRT3-KO mice fed with HFD have been shown to suffer from lipotoxicity (88). Together, these results suggest that SIRT3 may ameliorate cardiac lipotoxicity by modulating *de novo* fatty acid synthesis and β-oxidation. Overall, all findings reflect an integral regulatory role for SIRT3 in fatty acid catabolism in the heart (Figure 1).

## SIRT3 in Glucose Metabolism

Under physiological conditions, 20–30% of the cardiac energy demand is fulfilled by glucose and lactose metabolism (3). At the molecular level, glucose is transported into cardiomyocytes by glucose transporters GLUT-1 and GLUT-4. Mice with cardiac-specific knock-out of the glucose transporter GLUT-4 are characterized with compensatory cardiac hypertrophy, highlighting the importance of glucose metabolism in maintaining normal cardiac physiology (119).

Insulin sensitivity of the vasculature is central to the physiology of the heart, and is responsible for governing nutrient delivery to this omnivorous organ (120). Obesity is characterized with elevated levels of fasting blood glucose and insulin, and is associated with insulin resistance and endothelial dysfunction (120, 121). SIRT3 expression is downregulated in obesity. Studies in models of obesity reflect that SIRT3 can act as a positive regulator of insulin sensitivity in human and mice endothelial cells. In palmitate-treated insulin resistant endothelial cells, overexpression of SIRT3 promotes phosphorylation of key molecules in endothelial insulin signaling, namely, Akt and its downstream target endothelial nitric oxide synthase (eNOS). Consistent with this finding, *in vivo* experiments reflect exacerbated impairment of vasodilation, a function of endothelial NO production in obese SIRT3KO mice. Although the molecular mechanism for SIRT3 regulation of endothelial insulin sensitivity remains to be elucidated, this protective role of SIRT3 in obesity has been observed to be linked with reduced mitochondrial ROS production (67). Further, exploring insulin-dependent glucose uptake and utilization in this model can be expected to present interesting metabolic outcomes in the heart.

In addition to regulating endothelial insulin sensitivity, SIRT3 has also been implicated in the regulation of trans-endothelial glucose transport—a process that governs the availability of glucose for uptake and utilization by cardiomyocytes (122). Endothelial SIRT3-KO impairs expression of hypoxia-induced apelin, glucose transporters GLUT1 and GLUT4, and phosphofructokinase-2/fructose-2, 6-bisphosphatase-3 in endothelial cells—thereby disrupting glucose transport to cardiomyocytes (123). Reduced expression of endothelial GLUT receptors bears two significant consequences. Firstly, the reduction impairs endothelial glucose transport—impacting glucose concentration in the cardiac interstitial space, reducing glucose availability for cardiomyocytes. Secondly, impaired glycolysis is compensated with enhanced oxidative phosphorylation in the endothelium. Such metabolic reprogramming impairs angiogenesis and the microvascular function of endothelial cells, ultimately leading to heart failure (71).

In addition to glucose transport, mechanistic studies have revealed several molecular targets of SIRT3 for the regulation of glycolysis. Studies in cancer cells have revealed that SIRT3 negatively regulates hypoxia-inducible factor 1α (HIF-1α), a crucial transcription factor that induces glycolytic gene expression during hypoxia-induced metabolic reprogramming. In normoxia, SIRT3 suppresses HIF-1α by inhibiting the production of mitochondrial ROS, thereby attenuating ROS-mediated stabilization and activation of HIF-1α. Studies using models of cardiac hypertrophy show that SIRT3 inhibits ROS production by various means. Primarily, it activates ROS detoxifying enzyme manganese superoxide dismutase (MnSOD) *via* deacetylation. Simultaneously, it also activates transcription factor FOXO3a, which in turn upregulates expression of antioxidants manganese-dependent superoxide dismutase (MnSOD) and catalase (38, 124). In this manner, SIRT3 negatively regulates glycolysis under normoxic conditions.

SIRT3 is also known to regulate the activity of 2 vital glycolytic enzymes, hexokinase (HK) and phosphofructokinase (PFK). Studies in breast cancer have shown that SIRT3 might downregulate glucose metabolism by increasing cytosolic localization of Hexokinase II (HKII). While localization of HKII in the outer membrane of mitochondria favors glucose catabolism, cytosolic localization results in glucose anabolism (125). Localization of HKII to the mitochondrial outer membrane is mediated by its interaction with the voltage-dependent ion channel (VDAC)-adenine nucleotide translocator (ANT) complex. ANT located across the inner mitochondrial membrane, in turn, interacts with cyclophilin D (CypD) in the mitochondrial matrix. In skeletal muscles of HFD-fed SIRT3-KO mice, there is a marked increase in cytosolic localization of HKII, accompanied by impaired glucose metabolism. This result indicates that SIRT3 may play an essential role in forming the HKII-VDAC-ANT complex and subsequent activation of HKII to promote glycolysis in skeletal muscles (72, 126). Currently, in the heart, implications of SIRT3 mediated deacetylation of CypD have only been explored in the context of mitochondrial permeability membrane pore formation (127–129).

The next critical regulatory target of SIRT3 is phosphofructokinase (PFK). In cardiomyocytes, SIRT3 has been noted to enhance glucose metabolism by indirectly upregulating cardiomyocyte expression of 6-phosphofructo-2-kinase/fructose-2,6-bisphosphotase 3 (PFKFB3) *via* apelin (123). Furthermore, overexpression of SIRT3 has been shown to attenuate diabetic cardiomyopathy by deacetylating the transcription factor p53 and significantly reducing the expression of its downstream element, a fructose 2,6 bisphosphatase called TP53-induced glycolysis and apoptosis regulator (TIGAR) (47). Together, the downregulation of TIGAR and upregulation of PFKFB3 potentiate the marked increase in fructose 2,6 bisphosphate, a positive regulator of phosphofructokinase-1 (PFK1). These results suggest that SIRT3 may serve as a therapeutic target to rescue abnormal energetics under hyperglycaemic conditions (47).

SIRT3 is also a positive regulator of Pyruvate dehydrogenase complex (PDC), the enzyme complex that links glycolysis to the Krebs cycle. Under normal conditions, SIRT3 deacetylates pyruvate dehydrogenase phosphate-1 (PDP1) and pyruvate dehydrogenase E1 component subunit-α (PDHE1α) of the PDC to sustain physiological PDC activity (130). Furthermore, SIRT3 activates PDC by inhibiting HIF-1α. During hypoxia, HIF-1α transactivates PDC kinase, a negative regulator of PDC (131)—a function that is inhibited by SIRT3 under normal conditions. In this manner, SIRT3 positively regulates the flux of pyruvate into the tricarboxylic acid (TCA) cycle for glucose oxidation in the heart (Figure 1). Similarly, SIRT3 has also been reported as a positive regulator of glucose oxidation in skeletal muscles, where it deacetylates and activates PDC activity (48). Unlike in the heart, carbohydrates are the preferred energy substrate for skeletal muscles. During fasting, reduced SIRT3 levels result in hyperacetylation and decreased activity of PDC, which promotes glycolysis-glucose oxidation uncoupling and accumulation of pyruvate/ lactate. The glycolytic end products negatively regulate PFK1 and glucose utilization as an energy substrate (48). Similar to this, under conditions of ischemia and heart failure, uncoupling of glycolysis-glucose oxidation is observed in the heart. Moreover, NAD^+^ levels are depleted and the heart shows increased dependence on glycolysis for energy generation (132).

Interestingly, SIRT3-KO mice show increased fibrosis in several organs including the heart, lung, liver and kidney (62). Enhanced glucose metabolism, particularly glycolysis is essential for cardiac fibroblast activation and cardiac fibrosis (133, 134). Consistent with this, limiting glycolysis in heart has been shown to decrease cardiac fibrosis post myocardial infarction (134). The protective role of SIRT3 against kidney fibrosis under diabetic conditions is known to be mediated by suppression of HIF-1α and PKM2 dimer formation that upregulates expression of key glycolytic enzymes (135–137). Given that glycolysis has been linked with cardiac fibrosis, it would be interesting to explore the role of SIRT3 in conferring protection from cardiac fibrosis *via* regulation of glucose metabolism in the heart. Overall, the findings reflect an integral role for SIRT3 in regulation of glucose metabolism in the heart (Figure 1).

## Tricarboxylic Acid Cycle and Electron Transport Chain

Acetyl-CoA sourced from glucose and fatty acid metabolism enters the tricarboxylic cycle (TCA cycle) to generate NADH, FADH_2_, and CO_2_. SIRT3-KO mice are characterized by hyper-acetylation of enzymes involved in the tricarboxylic acid cycle (TCA) and the electron transport chain (ETC) (37, 68). Investigation using cardiac and extra-cardiac models has revealed several deacetylation targets of SIRT3 including citrate synthase (50), aconitase (51), isocitrate dehydrogenase (52), succinate dehydrogenase (39), and malate dehydrogenase (49, 138). Deacetylation is associated with increased enzyme activity of citrate synthase, isocitrate dehydrogenase, and succinate dehydrogenase. On the other hand, SIRT3 mediated deacetylation of aconitase and malate dehydrogenase results in downregulation of their enzyme activity (51).

In the ETC, succinate dehydrogenase of Complex II and the NDUFA9 subunit of complex I are targets of SIRT3 deacetylation. Hyperacetylation of NDUFA9 subunit in *Sirt*3^−/−^ mouse embryonic fibroblasts have been shown to strongly correlate with reduced oxidative phosphorylation (37). Additionally, studies performed in HEK293T have revealed human ATP synthase β as a deacetylation target of SIRT3 (139). Altogether, inhibiting SIRT3 expression culminates in a net reduction in oxidative phosphorylation—indicating that SIRT3 positively regulates oxidative phosphorylation under normal physiological conditions.

## SIRT3 in Ketone Metabolism

The heart is the largest consumer per body mass of ketones (4). Ketone bodies are synthesized in the liver and transported into target tissues by facilitated diffusion through MCT-1 transporters. Under physiological conditions, the heart oxidizes ketone bodies in proportion to their delivery. These enter the energy metabolism as acetyl-CoA, competing with acetyl-CoA generated from fatty acids and glucose metabolism for terminal oxidation (140, 141). Studies indicate that *Sirt*3^−/−^ mice display increased acetylation of hydroxy methyl glutaryl-CoA synthase (HMGS), an enzyme involved in ketogenesis in the liver. During fasting, deacetylation of HMGS increases its activity and, consequently, the levels of circulating ketone bodies (53). It is expected, although not established, that alteration in hepatic ketone metabolism may reflect a proportional change in cardiac ketone metabolism.

A recent study in cardiac hypertrophy reported SIRT3-dependent enhancement of ketone body metabolism *via* AMPK-mediated increase in the levels of monocarboxylic transporters 1 (MCT1) and 3-oxoacid CoA-transferase (OXCT1) (60). Another study reported that a key enzyme in ketogenesis, HMGS2 is dramatically upregulated in heart failure with preserved ejection fraction (HFpEF) (142). Its specific activity is, however, impaired in these hearts. HFpEF myocardium is also characterized by a net reduction in NAD^+^/NADH ratio and subsequently in SIRT3 expression. Since ketogenesis may serve as an essential energy source in failing hearts, SIRT3-dependent upregulation of ketogenesis may function as a “rescue strategy” in heart failure (142, 143).

## SIRT3 in Amino Acid Metabolism

The heart derives a marginal percentage of its energy from amino acid metabolism (4). SIRT3-mediated deacetylation results in glutamate dehydrogenase (GDH) activation, the enzyme responsible for converting Glutamine and Glutamate to TCA intermediate α-ketoglutarate (144). This regulation gains importance in ischemia when the TCA cycle intermediates are depleted. By increasing GDH activity, SIRT3 replenishes TCA intermediate levels *via* anaplerosis—thereby serving a cardioprotective function.

## Modulators of SIRT3

SIRT3 is downregulated under various pathological conditions. As detailed throughout the review, SIRT3 activation exhibits cardioprotective effects *via* remodeling of impaired cardiac metabolism. Modulating SIRT3 levels under various conditions may thus serve as a therapeutic strategy to ameliorate metabolism abnormalities.

Several plant metabolites protect against cardiovascular diseases by modulating SIRT3 activity. In *Sirt*3^−/−^ mice with heart failure, Resveratrol has been shown to ameliorate cardiac fibrosis by SIRT3-dependent inhibition of TGF-β/α-SMA signaling in heart failure (145). The dimerized form of Resveratrol, ε-viniferin, is also a known activator of SIRT3 (146, 147). Polydatin, a polyphenol isolated from *Polygonium cuspidatum* has been shown to ameliorate myocardial infarction in cardiomyocytes through a SIRT3-dependent increase in mitochondrial biogenesis and autophagy and a decrease in apoptosis (148). Dihydromyricetin from *Ampelopsis grossedentata* has been shown to reduce cardiac ischemia reperfusion injury by improving mitochondrial function and reducing oxidative stress in a SIRT3-dependent manner (149). Berberine and Honokiol are also known activators of SIRT3. They have independently been shown to protect the heart against doxorubicin, an antineoplastic drug that induces cardiotoxicity. They confer protection from doxorubicin-induced cardiomyopathy by preventing oxidative damage, mitochondrial dysfunction, and cell death (150, 151).

Yet another known SIRT3 activator, Salidroside, extracted from *Rhodiola rosea* has been shown to protect against cardiac dysfunction in animal models of diabetes and myocardial infarction (MI) (152, 153). In mice models of MI, Salidroside reduces fibrosis and infarct size and improves cardiac function. However, the role of SIRT3 activation in this process remains to be understood. In the mouse model of diabetes, salidroside is known to confer protection by increasing SIRT3 expression and translocation to mitochondria, promoting MnSOD activity, thereby reducing oxidative damage observed in diabetic patients cardiomyopathy (154). Another activator of SIRT3 is Licoisoflavone A, a naturally occurring flavonoid and active ingredient of Tongmaiyangin, a Chinese therapeutic pill composed of 11 herbs. It has been shown to inhibit angiotensin II-induced cardiac hypertrophy *via* SIRT3 activation (155). Most recently, Quercetin was identified as SIRT3 activator. It was shown to preserve mitochondrial function and structure, ameliorate cardiac hypertrophy, and improve overall cardiac function *via* activation of the SIRT3 in spontaneously hypertensive rats (156).

Several small molecules have also been identified as SIRT3 activators. Depletion of NAD^+^, the Sirtuin cofactor, is a major contributor to various cardiac pathologies. Subsequently, NAD^+^ repletion has proved to be effective in amelioration of these pathologies conditions (157, 158). Administration of exogenous NAD^+^ in mice and *in vitro* models of hypertrophy has been shown to confer cardioprotection from hypertrophy in a SIRT3-dependent manner. Exogenous NAD^+^ activates SIRT3, which in turn deacetylates and activates LKB1 kinase, thereby promoting anti-hypertrophic LKB2-AMPK signaling. In addition to this, SIRT3 activation can also be expected to block pro-hypertrophic Akt1 signaling by inhibiting ROS production (105). Early studies also demonstrated the use of the NAD^+^ biosynthetic precursor nicotinamide riboside to enhance SIRT3 activity in mouse embryonic fibroblasts (MEFs), as indicated by reduced acetylation of SIRT3 targets in these cells (159). Most recently, oral administration of nicotinamide riboside in HFpEF mice was noted to result in reversal of the heart failure phenotype and recovery of mitochondrial function (158). Although the SIRT3 protein expression in these mice remains unaltered, nicotinamide riboside may be expected to serve as a SIRT3 activator by enhancing its deacetylation activity without altering its expression. However, this possibility remains to be tested. Another prominent small molecule known to activate SIRT3 in the heart is NAD^+^ precursor nicotinamide mononucleotide (NMN). Using a SIRT3-KO/Friedreich's ataxia cardiomyopathy (FXN)-KO double knockout model, a study demonstrated that NMN administration restores cardiac energetics and function in these hearts a SIRT3-dependent manner (160).

Other small molecule activators of SIRT3 include Metformin, which is commonly used in the treatment of type 2 diabetes. Metformin augments SIRT3, thereby improving heart failure post-myocardial infarction by enhancing cardiac metabolism and reducing apoptosis (90). The hormone, melatonin has been shown to ameliorate IR injury by increasing the expression and activity of SIRT3 levels (161). Choline, a precursor of the neurotransmitter acetylcholine, is also known to improve diabetic cardiomyopathy through SIRT3 mediated enhancement of mitochondrial protein unfolded response, fatty acid and ketone body metabolism (60). Elabela, a small endogenous peptide, is shown to protect against diabetic cardiomyopathy by inhibiting oxidative stress and apoptosis *via* SIRT3 mediated deacetylation of the transcription factor FOXO3a (162).

All phytochemical and small molecule activators are summarized in Table 3. It is worthwhile to note that the unavailability of proven pharmacological activators of SIRT3 currently remains a challenge in exploiting the therapeutic potential of SIRT3 regulation in cardiac metabolism. Studies characterizing SIRT3 activators are riddled with mechanistic and methodological gaps that prevent them from being translated for clinical use. Most studies proposing the use of phytochemicals as SIRT3 activators are correlation-based, and fail to demonstrate direct binding to, or mechanism of indirect activation of SIRT3 by the modulator. Most of these studies also lack the use of a rescue model to establish SIRT3 activation as the mechanism underlying cardio-protection against various diseases. Moreover, the therapeutic potential of many of these phytochemicals remains to be explored *in vivo* in models of cardiac pathologies such as heart failure. Furthermore, they fail to evaluate the effect of modulator treatment on cardiac function—which is an essential parameter for exploring pharmaceutical potential of a proposed therapeutic. Finally, and most importantly, it is unclear whether these compounds are selective activators of SIRT3. For instance, in addition to SIRT3, resveratrol can modulate several other molecules, including SIRT1 (166–168), SIRT5 (169), certain kinases and ATP synthase (170). Similarly, NAD^+^ also serves as a substrate for enzymes involved in calcium signaling and DNA damage repair (157). This characterizes these activators with pleiotropic effects and renders them less suitable for pharmacological use.

**Table 3:** Activators of SIRT3.

**SIRT3 activator**	**Source**	**Model system**	**Physiological effects of activator treatment**	**Cardiac phenotype**	**References**
**Phytochemicals**					
Resveratrol	*Vitis vinifera, Morus rubra, Vaccinum* spp., *Polygonum cuspidatum, Artocarpus* sp., *Rheum raponticum, Pinus sylvestris, Cassia* sp., *Arachis hypogea, Picea* sp.	Mice with cardiac hypertrophy	TGF-β/α-SMA signaling (↓)	Cardiac fibrosis (↓)	(145, 163)
Polydatin	*Polygonium cuspidatum*	Mice with myocardial infarction	Mitochondrial biogenesis (↑), autophagy (↑), apoptosis (↓)	Cardiac function (↑)	(148)
Dihydromyricetin	*Ampelopsis grossedentata*	Mice with myocardial ischemia/reperfusion	Mitochondrial function (↑), Oxidative stress (↓)	Cardiac IR injury (↓)	(149)
Berberine	*Berberis vulgaris*	Doxorubicin-treated cardiomyocytes	Mitochondrial biogenesis (↑), Mitochondrial fragmentation (↓), Oxidative stress (↓), Apoptosis (↓)	DOX-induced cardiotoxicity (↓)	(150)
Honokiol	*Magnolia officinalis*	Doxorubicin-treated mice hearts	Mitochondrial function (↑), Mitochondrial DNA damage (↓), Oxidative stress (↓), Apoptosis (↓)	DOX-induced cardiotoxicity (↓)	(38, 151, 164)
Salidroside	*Rhodiola rosea*	HFD + Streptozocin-induced diabetic mice; High fat and High glucose-conditioned neonatal rat cardiomyocytes	AMPK activity (↑), PGC-1α expression (↑), Mitochondrial mass (↑), Mitochondrial superoxide production (↓)	Cardiac fibrosis (↓), Cardiac function (↑)	(154)
Licoisoflavone A	*Glycyrrhiza uralensis*	Phenylephrine (PE)-induced hypertrophy in neonatal rat cardiomyocytes	ANF and BNP expression (↓)	PE-induced hypertrophy (↓)	(155)
Quercetin	*Morus alba, Moringa oleifera, Brassica* sp., *Prunus domestica* etc.	Angiotensin II-induced hypertrophy in cardiomyocytes	Mitochondrial function (↑), Oxidative stress (↓)	Angiotensin II-induced cardiac hypertrophy (↓)	(156, 165)
**Other molecules**					
Exogenous NAD^+^		Isoproterenol-induced cardiac hypertrophy in mice; Phenylephrine (PE)-induced hypertrophy in neonatal rat cardiomyocytes	LKB1-AMPK signaling (↑), *Anf* expression (↓), *Collagen*-α expression (↓)	Cardiac fibrosis (↓), Isoproterenol-induced cardiac hypertrophy (↓), Cardiac function (↑)	(105)
Nicotinamide mononucleotide (NMN)		Friedreich's ataxia cardiomyopathy (FXN) mouse model	Glycolytic flux (↓)	Cardiac function (↑)	(160)
Metformin		Mice with myocardial infarction	Mitochondrial function (↑), PGC-1α activity (↑), Apoptosis (↓)	Cardiac function (↑)	(90)
Melatonin		Mice with myocardial ischemia/reperfusion, H9C2 cardiomyocytes	Apoptosis (↓), Oxidative stress (↓)	Infarct size (↓), Post-ischemic contractile function (↑)	(161)
Choline		Abdominal Aortic Banding (AAB) rats; Angiotensin II-induced hypertrophy in neonatal rat cardiomyocytes	AMPK activity (↑), mtUPR (↑), Mitochondrial function (↑), Ketone body metabolism (↑), BNP expression (↓)	Cardiac function (↑)	(60)
Elabela		Streptozotocin-induced type I diabetic mouse model	SOD-2 and MnSOD expression (↑), Apoptosis (↓), Oxidative stress (↓), Interstitial collagen deposition (↓)	Cardiac fibrosis (↓), Cardiac function (↑)	(162)

Overall, these gaps warrant the need for comprehensive studies to better characterize SIRT3 activators, and identify suitable candidates for clinical studies to explore their therapeutic potential.

## Summary and Future Perspective

Metabolic dysfunction is a common feature of cardiac pathologies like hypertrophy, ischemic reperfusion injury, and heart failure. Therefore, understanding the regulation of myocardial metabolism is of keen interest in identifying therapeutic targets for cardiac pathologies treatment. SIRT3 appears as a promising target for improving myocardial metabolism due to its proximity to the mitochondrial metabolic machinery and the cardiac pump, along with its functional dependence on the cellular [NAD^+^]/[NADH] ratio. Recent studies have highlighted the regulatory roles of SIRT3 in physiological and pathological cardiac metabolism. Overall, SIRT3 presents as a positive regulator of cardiac energy metabolism. It has been shown to enhance glucose and fatty acid oxidation and promote ketogenesis for energy generation in the heart. SIRT3 expression is downregulated in models of cardiac hypertrophy, heart failure, ischemia, and diabetic cardiomyopathy. Activating SIRT3 in these hearts ameliorates metabolic dysfunction, thereby attenuating the damage associated with pathological metabolic reprogramming. Implications of SIRT3-mediated metabolic regulation need to be studied to identify SIRT3 modulators for the treatment of these numerous cardio pathologies.

Metabolic targets of SIRT3 have been studied extensively in extra-cardiac tissues, especially in the liver and skeletal muscles. The heart, however, differs vastly in its use of metabolic substrates from other organs. It is uniquely omnivorous and relies primarily on fatty acids for energy generation. Thus, it is expected that metabolic targets and mechanisms of SIRT3 regulation may differ in the heart. It would be of keen interest to understand if and how SIRT3 targets identified in extra cardiac tissues are regulated in the heart and identify novel targets specific to cardiac metabolism.

Furthermore, the heart is composed of multiple cell types, including fibroblasts, cardiomyocytes, smooth muscle cells, and endothelial cells. Each cell type has a distinct metabolic profile. It would be fascinating to study the role of SIRT3 in each cell type in the heart using cell-type-specific transgenic or knock-out models of SIRT3. It would highlight how SIRT3 regulates metabolic crosstalk between different cell types to coordinate overall cardiac energetics. Moreover, since the heart is a highly metabolic organ, studying novel routes of SIRT3 regulation in the cardiac context may reveal exciting insights into the role of SIRT3 as a regulator of whole-body energetics.

## Author Contributions

NS planned the project, oversaw the work, secured funding, and prepared the final document for submission. KM and AM wrote the review, prepared figures, and were also involved in the planning. All authors contributed to the article and approved the submitted version.

## Funding

This work was supported by research funding from the Department of Science and Technology and the Department of Biotechnology, Government of India.

## Conflict of Interest

The authors declare that the research was conducted in the absence of any commercial or financial relationships that could be construed as a potential conflict of interest.

## Publisher's Note

All claims expressed in this article are solely those of the authors and do not necessarily represent those of their affiliated organizations, or those of the publisher, the editors and the reviewers. Any product that may be evaluated in this article, or claim that may be made by its manufacturer, is not guaranteed or endorsed by the publisher.
